# Single-Cell RNA-Sequencing Identifies Infrapatellar Fat Pad Macrophage Polarization in Acute Synovitis/Fat Pad Fibrosis and Cell Therapy

**DOI:** 10.3390/bioengineering8110166

**Published:** 2021-10-29

**Authors:** Dimitrios Kouroupis, Thomas M. Best, Lee D. Kaplan, Diego Correa, Anthony J. Griswold

**Affiliations:** 1 Department of Orthopedic Surgery, UHealth Sports Medicine Institute, University of Miami, Miller School of Medicine, Miami, FL 33146, USA; txb440@med.miami.edu (T.M.B.); kaplan@med.miami.edu (L.D.K.); dxc821@med.miami.edu (D.C.); 2 Diabetes Research Institute and Cell Transplant Center, University of Miami, Miller School of Medicine, Miami, FL 33136, USA; 3 John P. Hussman Institute for Human Genomics, University of Miami, Miller School of Medicine, Miami, FL 33136, USA; agriswold@med.miami.edu; 4 The Dr. John T. Macdonald Foundation Department of Human Genetics, University of Miami, Miller School of Medicine, Miami, FL 33136, USA

**Keywords:** infrapatellar fat pad, single-cell RNA-sequencing, macrophages, mesenchymal stem cells, inflammation

## Abstract

The pathogenesis and progression of knee inflammatory pathologies is modulated partly by residing macrophages in the infrapatellar fat pad (IFP), thus, macrophage polarization towards pro-inflammatory (M1) or anti-inflammatory (M2) phenotypes is important in joint disease pathologies. Alteration of M1/M2 balance contributes to the initiation and progression of joint inflammation and can be potentially altered with mesenchymal stem cell (MSC) therapy. In an acute synovial/IFP inflammation rat model a single intra-articular injection of IFP-MSC was performed, having as controls (1) diseased rats not receiving IFP-MSC and (2) non-diseased rats. After 4 days, cell specific transcriptional profiling via single-cell RNA-sequencing was performed on isolated IFP tissue from each group. Eight transcriptomically distinct cell populations were identified within the IFP across all three treatment groups with a noted difference in the proportion of myeloid cells across the groups. Largely myeloid cells consisted of macrophages (>90%); one M1 sub-cluster highly expressing pro-inflammatory markers and two M2 sub-clusters with one of them expressing higher levels of canonical M2 markers. Notably, the diseased samples (11.9%) had the lowest proportion of cells expressing M2 markers relative to healthy (14.8%) and MSC treated (19.4%) samples. These results suggest a phenotypic polarization of IFP macrophages towards the pro-inflammatory M1 phenotype in an acute model of inflammation, which are alleviated by IFP-MSC therapy inducing a switch towards an alternate M2 status. Understanding the IFP cellular heterogeneity and associated transcriptional programs may offer insights into novel therapeutic strategies for disabling joint disease pathologies.

## 1. Introduction

The knee infrapatellar fat pad (IFP) and synovium play a key role in modulating the inflammatory response of joint disorders and are considered a single anatomical and functional unit [1], exhibiting molecular crosstalk important in healthy joint function as well as the onset and progression of inflammatory joint disease [2,3,4]. In particular, these tissues are increasingly recognized to orchestrate the local immune and inflammatory responses observed in early-stage osteoarthritis (OA) [2,3,4,5,6,7,8,9,10]. The IFP serves as a site of immune cell infiltration and is the origin of pro-inflammatory and articular cartilage catabolic mediators (e.g., IFN-γ, TNF-α, IL-1,-6,-8, and metalloproteinases), and is a source of the pain-transmitting [11,12,13,14] and immune/inflammation modulatory [15,16,17,18] mediator Substance P. Consequently, the IFP and synovium undergo cycles of inflammation inducing progressive fibrotic changes, supporting synovitis and IFP fibrosis as key elements during the onset and progression of joint disease [2,3,4,5,6,7,8,9,10], particularly in those conditions possessing an inflammatory phenotype [19,20]. On this basis, the IFP is a potential therapeutic target to mitigate synovitis/IFP fibrosis incidence and progression [9,21,22]. Therefore, an IFP-based therapeutic strategy aimed at restoring joint homeostasis could potentially mitigate progression of synovitis/IFP fibrosis-related disabling pain and joint degeneration.

Homeostasis of all tissues depend on molecularly and functionally fine-tuned cell types that upon injury and/or disease may adopt alternative phenotypes leading to pathological conditions. This is evident in the IFP that is mainly composed of adipocytes responsible for the IFP’s metabolism [21,22,23,24], stromal cells, and immune-related cells including mast cells, natural killer, T and B cells, and importantly monocytes/macrophages [25,26]. The latter are key components of the innate immune system possessing important effector functions including actively participating in tissue homeostasis [27] and inflammatory pathological processes [28,29]. Upon activation of the inflammatory cascade, macrophages can instruct the responses of the adaptive immune system (e.g., T cells) by becoming polarized towards classically activated pro-inflammatory (M1) or alternatively activated M2 anti-inflammatory phenotypes by a variety of signaling mediators (e.g., interleukins and interferons) secreted within the IFP niche (reviewed in [22]). Dysregulation of the M1/M2 phenotypic balance of synovial and IFP macrophages may account for triggering and further progression of joint pathologies by helping to maintain a sustained low-grade inflammatory state [28,29].

Mesenchymal Stem Cells (MSC), originally described as progenitors of mesodermal tissues, can exert “medicinal signaling” activities within sites of injury [30]. This signaling includes modulation of local immune responses and trophic effects, which ultimately induce the restoration of local tissue homeostasis [31]. MSC-based therapy for joint pathologies has advanced to promising clinical trials [32,33,34] due to their immunomodulatory, anti-inflammatory, anti-fibrotic properties in part through direct effects on monocyte/macrophage phenotypic polarization [35,36]. This MSC–macrophage crosstalk is well established by previous studies indicating that MSC can polarize pro-inflammatory (M1) macrophages into an M2 anti-inflammatory phenotype through a PGE2-dependent mechanism [21,37,38]. Importantly, we have recently reported in a rat model of synovitis and IFP fibrosis that a single intra-articular injection of BM- or IFP-derived MSC reverses inflammation and fibrosis linked with polarization from an M1 enrichment in disease to a more M2 phenotype following MSC therapy [26,39,40]. On this basis, recent single-cell RNA sequencing analysis indicated that adipose-derived MSC (which are phenotypically and molecularly similar to IFP-MSC) show less transcriptomic heterogeneity and superior capacity in regulating inflammation compared to bone marrow-derived MSC [41]. This finding further supports our selection to use IFP-MSC injections for the treatment of joint immune and inflammatory responses.

Despite the encouraging observations noted above, several questions remain regarding the role of IFP macrophages, their possible phenotypes beyond the established M1 and M2, their role in inflammatory joint disease, and the effects of locally injected MSC on their molecular and functional phenotypes. To help address these lingering questions, herein we present a single cell transcriptional characterization of the rat IFP contrasting its cellular composition and gene expression in health, during synovitis/IFP fibrosis, and after MSC therapy.

Understanding the IFP’s cellular heterogeneity and associated transcriptional programs across homeostasis, disease, and post-therapy, not only helps elucidate the dynamic cellular programs involved in joint pathological conditions, but also offer insights into the identification of potentially novel treatment strategies. Future innovative joint cellular therapies will be able to incorporate emerging notions such as the role of IFP inflammation/fibrosis as initiating pathological events [11,17], the roles of pro-inflammatory resident M1 macrophages as amplifiers of the molecular cascades that lead to altered joint homeostasis [28,29], and most critically, how to revert those cellular changes to potentially mitigate disease progression.

## 2. Materials and Methods

### 2.1. Cell and Animal Protocols

IFP-MSC were isolated from IFP tissue obtained from two de-identified, non-arthritic human patients (one female (IFP-MSC1) and one male (IFP-MSC2), both 32 years old, BMI: 18.5–24.9 (normal or healthy weight)) undergoing elective knee arthroscopy (ACL reconstruction and/or meniscal repair) at the Lennar Foundation Medical Center, University of Miami after providing written informed consent. All procedures were carried out in accordance with relevant guidelines and regulations and following a protocol determined by the University of Miami IRB not as human research (based on the nature of the samples as discarded tissue).

The animal protocol was approved by the Institutional Animal Care and Use Committee (IACUC) of the University of Miami, USA (approval no. 16-008-ad03) and conducted in accordance with the ARRIVE guidelines [42]. Twelve (#12) Sprague Dawley rats (all male; mean weight 400 g) were used. The animals were housed to acclimate for 1 week before the initiation of experiments. One rat was housed per cage in a sanitary, ventilated room with controlled temperature, humidity, and under a 12/12 h light/dark cycle with food and water provided ad libitum.

### 2.2. Mono-Iodoacetate Model of Acute Synovial/IFP Inflammation

Acute synovial/IFP inflammation was generated by intra-articular injection of 1 mg of mono-iodoacetate (MIA) in 50 µL of saline in rat knees. Under isoflurane inhalation anesthesia, rat knees were flexed 90° and MIA was injected into the medial side of the joint with a 27G needle using the patellar ligament and articular line as anatomical. This short exposure to MIA for both the diseased and treated groups has been shown to induce inflammatory changes within the synovium and adjacent IFP [43]. In total, three groups were tested: treated, diseased, and healthy (6 animals/group, 12 knees/group: statistical significance of 0.05 with 80% power). For the treated group, at day 4 a single intra-articular injection of 500,000 IFP-MSC in 50 µL of Euro-Collins solution (MediaTech, Houston, TX, USA) was performed into the right knee only (IFP-MSC1 and IFP-MSC2 Treated groups). For the diseased group, in the same rats left knees received only MIA but not IFP-MSC, as a negative control to avoid intra-animal variation. The healthy group consisted of six rats that did not receive any injections. All animals were sacrificed 4 days after IFP-MSC injection (day 8 total).

### 2.3. Tissue Preparation

Rat knee joints were harvested by cutting the femur and tibia/fibula 1 cm above and below the joint line, muscles were removed, and fat pads were carefully mechanically dissected from each knee by totally removing all surrounding tissues including the synovium. Upon extraction, IFPs were pooled together to generate three separate groups: Healthy, Diseased, and IFP-MSC treated, whereas each group contained two replicates with 6 IFPs each. Pooled groups were washed repeatedly with PBS (Sigma Aldrich, St. Louis, MO, USA), followed by enzymatic digestion using 235 U/mL Collagenase I (Worthington Industries) diluted in PBS and 1% bovine serum albumin (Sigma Aldrich) for 2 h at 37 °C with agitation. Cell digests were inactivated with DMEM + 10% FBS, washed, and directly proceeded to RNA extraction for single-cell RNA-sequencing.

One fat pad per group was fixed with 10% neutral buffered formalin (Sigma Aldrich) for 10 minutes at room temperature, embedded in parrafin, and serial 4 μm sections were obtained. Hematoxylin and Eosin (H&E) staining was performed to evaluate the structure and morphology of fat pads. Microscope images of cytochemically stained tissues were acquired using 10× objectives Leica DMi8 microscope with Leica X software (Leica, Buffalo Grove, IL, USA). Individual H&E 10× images were imported to ImageJ software and after color deconvolution the threshold range was set up to 100–144. Parameters included in the analysis were area, area fraction, limit to threshold, display label. Based on histochemical stainings, tissue synovitis/fibrosis was evaluated in 4 sections per fat pad and 6 microscopy fields per section.

### 2.4. Single-Cell RNA-Sequencing

Single-cell RNA-sequencing was performed in the Center for Genome Technology at the HIHG. Cells at a concentration of 1200 cell/μL were loaded on the 10× Genomics Chromium platform to isolate ~5,000 nuclei per sample and create individually barcoded Gel bead-in-Emulsions (GEMs) which were processed using the Chromium Single Cell 3′ Reagent Version 3 Kit. Sequencing libraries were evaluated for quality on the Agilent Tape Station (Agilent Technologies, Palo Alto, CA, USA) and quantified using a Qubit 2.0 Fluorometer (Invitrogen, Carlsbad, CA, USA) and qPCR before sequencing on the Illumina NovaSeq 6000 targeting 100,000 reads per cell with sequencing parameters: Read1, 28 cycles; Index1, 8 cycles; Read2, 98 cycles.

### 2.5. Single-Cell RNA-Sequencing Data Analysis

Primary bioinformatics analysis was performed with the 10X Genomics CellRanger v3.0.2 software followed by secondary analysis with the Seurat 3.1 pipeline [44]. Briefly, following quality control for total detected genes and mitochondrial genes and doublet removal we integrated all sample replicates and performed normalization and data scaling followed by cell cluster identification. Canonical markers for each cluster were identified using a differential expression meta-analysis between each cluster against all others and then assigned identity using published transcriptomic characterizations of cell types. Differential expression between clusters was determined by MAST test which uses a generalized linear model framework using cell detection rate within replicates and across groups as a covariate [45]. MAST has low error and false discovery rates in comparison with other single cell differential expression methods [46]. Differentially expressed genes were tested for enrichment in Gene Ontology Biological Processes using DAVID [47,48]. Detailed single cell data processing steps are available in the Supplementary methods.

### 2.6. M1/M2 Macrophages Immunolocalization 

For CD86 (M1) and CD206 (M2) immunofluorescence staining, sections were incubated with 1× citrate buffer solution at 60 °C overnight for antigen retrieval, permeabilized with 1× PBS + 0.2% Triton X-100 for 20 minutes at room temperature, and incubated with blocking buffer (1× PBS + 0.1% Triton X-100 with 10% rabbit serum) for 1 h at room temperature. In between different treatments, sections were washed with 1× PBS. Mouse anti-rat CD86 monoclonal antibody (clone BU63, cat. ab213044, Abcam, Waltham, MA, USA) was prepared in blocking buffer (1:1000) and rabbit anti-rat CD206 polyclonal antibody (cat. Ab64693, Abcam) was prepared in blocking buffer (1:250). Sections were incubated with CD86 and CD206 at 4 °C overnight. Sections were washed with 1× PBS + 0.01% Triton X-100 and incubated for 1 h with secondary antibodies Alexa Fluor488 conjugated goat anti-mouse IgG antibody (cat. A32723) and AlexaFluor647 conjugated goat anti-rabbit IgG antibody (cat. A32733) (both from Invitrogen, ThermoFisher Scientific, Waltham, MA, USA) prepared in blocking buffer (1:400) at room temperature. Controls were incubated with secondary antibodies only. All sections were rinsed with 1× PBS, mounted in prolong gold antifade reagent with DAPI (Invitrogen), and microscope images were acquired using 20× objective Leica DMi8 microscope with Leica X software (Leica). M2/M1 ratio was evaluated in 4 sections per fat pad and 6 microscopy fields per section with ImageJ software.

### 2.7. Statistical Analysis 

Normal distribution of values was assessed by the Kolmogorov–Smirnov normality test. Statistical analysis was performed using non-parametric Kruskal–Wallis test and one-way ANOVA for multiple comparisons. Tests were performed with GraphPad Prism v7.03 (GraphPad Software, San Diego, CA, USA). Differences in cell type proportions between groups were tested using a two-proportions z-test. Statistical analyses were performed using the “catfun” package in the R computing environment. Level of significance was set at *p* < 0.05.

### 2.8. Supplementary Materials and Methods

#### 2.8.1. MSC Cell Isolation and Expansion

IFP tissue (5–10 cc) was mechanically dissected and washed repeatedly with Dulbecco’s Phosphate Buffered Saline (PBS; Sigma), followed by enzymatic digestion using 235 U/mL Collagenase I (Worthington Industries, Columbus, OH, USA) diluted in PBS and 1% bovine serum albumin (Sigma) for 2 h at 37 ℃ with agitation. Cell digests were inactivated with complete media [DMEM low glucose GlutaMAX (ThermoFisher Scientific, Waltham, MA, USA) + 10% fetal bovine serum (FBS; VWR, Radnor, PA, USA)], washed, and seeded at a density of 1 × 10^6^ cells/175 cm^2^ flask in complete human platelet lysate (hPL) medium. Complete hPL medium was prepared by supplementing DMEM low glucose GlutaMAX with hPL solution and 0.024 mg/mL xeno-free heparin (PL Bioscience, Aachen, Germany) to obtain a 10% hPL final concentration. IFP-MSC were cultured at 37 °C 5% (*v/v*) CO_2_ until 80% confluent as passage 0 (P0), then passaged at a 1:3 ratio until P2, detaching them with TrypLE™ Select Enzyme 1× (Gibco, ThermoFisher Scientific, Waltham, MA, USA) and assessing cell viability with 0.4% (*w/v*) Trypan Blue (Invitrogen, ThermoFisher Scientific).

#### 2.8.2. Single-Cell RNA-Sequencing Analysis

We used the 10X Genomics CellRanger v3.0.2 software to create de-multiplexed FASTQ files from the raw sequencing output with the {mkfastq} command followed by mapping of reads and quantification of gene expression using a customized human (GRCh38)—rat (Rnor6.0) reference genome with the {count} command and found less than 1% of cells mapping to the human reference. Thus, we repeated the {count} command aligning only to the rat reference genome. CellRanger performs an initial quality control step separating true cells from empty droplets using the EmptyDrops algorithm [49]. Cells passing this initial calling QC in CellRanger were analyzed further with the Seurat 3.1 pipeline [44] implemented in R v3.6.1 and RStudio v1.2.1335_64x for data filtering, normalization, integration, and downstream analysis.

We calculated the number of unique cell barcodes identifiers, number of genes expressed, and number of reads mapping to mitochondrial genes for all cells passing CellRanger quality control in each of the six sample replicates independently. We removed poor quality cells and potential doublets by excluding those outside the 5th and 95th percentile of the number of genes and the number of reads per gene. This has shown to be a useful threshold in recent sequencing studies [50,51]. Furthermore, we excluded cells with greater than 10% mitochondrial reads to exclude low quality or dying nuclei which tend to carry more mitochondrial RNA. From this set of cells, we ran DoubletFinder [52] for removal of other predicted doublets.

#### 2.8.3. Single Nucleus RNA-Sequencing Data Integration 

Following quality control, global normalization was performed on all cells from each sample independently in Seurat to normalize gene expression of each cell by total expression per sample, followed by log transformation. We identified the 2000 most variable genes in each sample and performed data integration across these to identify shared cell expression profiles across all sample replicates followed by identification of anchor genes and data integration using following a recently published protocol [53]. Finally, global-scaling of the integrated data set to remove batch effects and unwanted sources of biological variation was performed on the integrated dataset.

#### 2.8.4. Single Nucleus Clustering

The scaled integrated dataset was used for further downstream processing to identify common cell types and enable comparative analyses across the samples. First, 50 principal components were calculated on the scaled data and the PCs were projected into two dimensions using the UMAP algorithm [54]. Similar cells were clustered from the principal components and clusters defined using a resolution in Seurat of 0.05 resulting in seven distinct clusters. Finally, we identified the canonical marker genes for each cluster across samples by performing differential gene expression between each cluster and every other cluster and combining the p-values using meta-analysis methods from the R package MetaDE [55]. Clusters were assigned a cell type based on previously published data on RNA sequencing transcriptomic profiling.

## 3. Results

### 3.1. IFP-MSC Effectively Reverse IFP Fibrosis

Herein, using our established IFP-MSC manufacturing protocol [40,56], we investigated the effects of IFP-MSC therapeutic treatment on the IFP tissue cellular heterogeneity.

**Figure 1:**
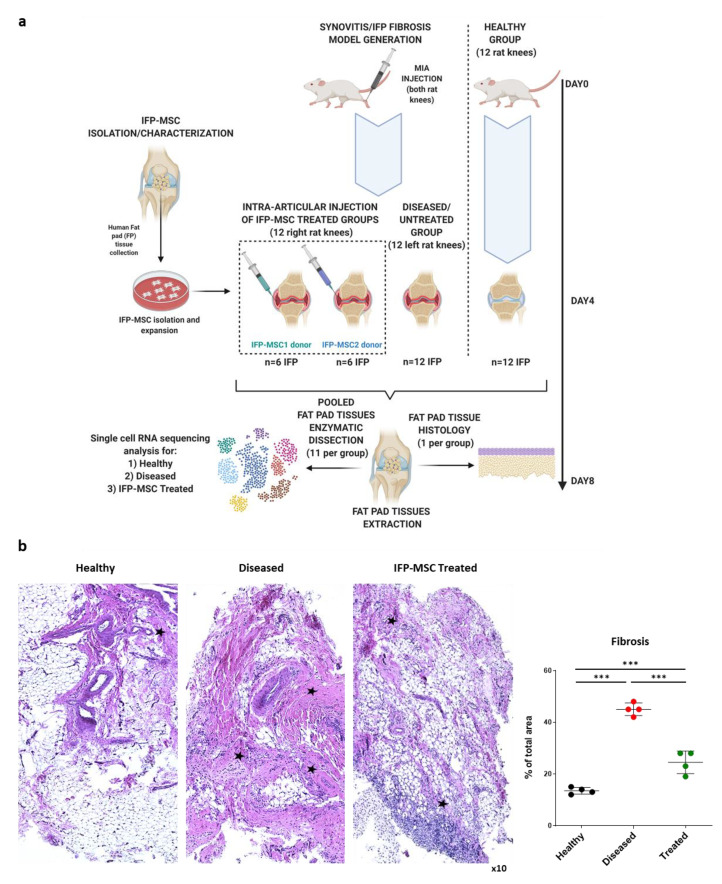
A rat model of induced acute synovitis and IFP fibrosis was used to understand IFP cellular heterogeneity and associated transcriptional programs. (**a**) Single-cell RNA-sequencing workflow for three groups (healthy, diseased, IFP-MSC treated). (**b**) H&E staining of IFP tissue revealed significant (*p* < 0.05) reduction in IFP fibrosis 4 days after IFP-MSC administration (marked with asterisks) (*** *p* < 0.001). Statistical analysis was performed using non-parametric Kruskal–Wallis test.

Specifically, a rat model of induced acute synovitis and IFP fibrosis was used to understand IFP cellular heterogeneity and associated transcriptional programs across homeostasis, disease, and after IFP-MSC treatment at the single cell level (Figure 1a). Compared with diseased animals, all animals that received IFP-MSC showed a significant reduction in IFP fibrosis 4 days after their administration (Figure 1b, marked with asterisks). Specifically, single IFP-MSC intra-articular injection resulted in a significant (*p* < 0.05) 54% reduction of IFP fibrosis compared to the diseased group (24.5 ± 4.3% vs. 45 ± 2.4%), reaching comparable levels to the healthy rat group (13.5 ± 1.3%). Day 4 after IFP-MSC administration was selected for fat pad tissue interrogation as according to our experience using the MIA model [26,40], this is the timepoint where we observe a striking spatial inverse correlation between IFP-MSC presence and reversal of signs of synovitis and IFP fibrosis.

### 3.2. Characterization of Cellular Make-Up of IFP with Single-Cell RNA-Sequencing

**Figure 2:**
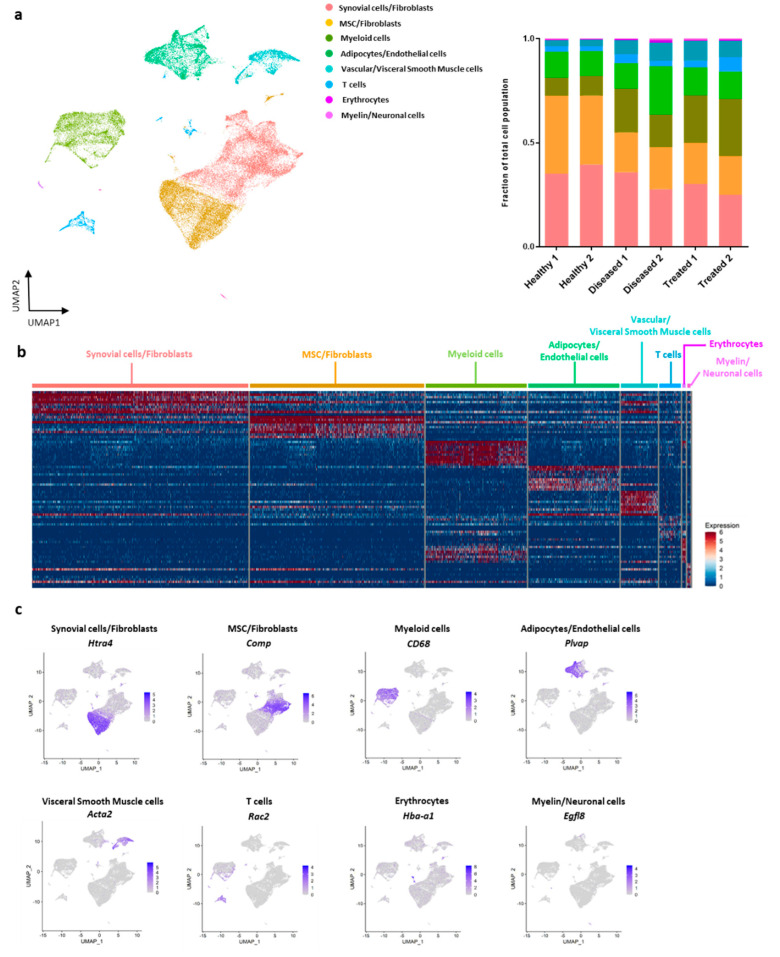
Single-cell RNA-sequencing clustering and analysis of IFP tissue. (**a**) Eight transcriptionally distinct cell populations were identified, with myeloid cells being one of the most prominent within the IFP tissue. Each point represents a single cell and separate subpopulations identified were color-coded. Cell population proportions revealed higher presence of myeloid cells within the IFP tissue in the MSC treated samples. Cell population proportions per sample were visualized by stacked bars plot. Healthy 1 and Healthy 2: healthy group replicates consisting of six rat knees each; Diseased 1 and Diseased 2: diseased group replicates consisting of six rat knees each; Treated 1 and Treated 2: treated group replicates (one injected with IFP-MSC1 and one injected with IFP-MSC2) consisting of six rat knees each. (**b**) Heatmap of top 10 marker genes defining each cell type. Each column represents a cell and each row a gene. High expression is red and lower expression is blue. (**c**) Gene localization plots identified specific canonical marker genes related to distinct cell populations. Gene expression levels depicted from gray (low) to purple (high).

Intact fat pad tissues originated from Healthy, Diseased, and IFP-MSC treated groups were enzymatic digested and corresponding cell digests were directly processed for RNA extraction and single-cell RNA-sequencing. After quality control, we obtained data from a total of 38,432 total nuclei (4329–9921 nuclei per replicate), sequenced at a median depth of ~87,000 reads per cell with on average ~1700 genes/cell. Initial alignment to the human genome revealed less than 1% of total reads mapping uniquely to the human genome, thus we proceeded with analysis of only rat genes. There was no significant difference between groups in terms of percentage of aligned reads (84.8 ± 0.1%, 82.3% ± 0.6%, and 82.9% ± 0.1% of reads mapping uniquely) to the Rnor6.0 genome in healthy, diseased, and treated groups, respectively. A two-dimensional UMAP plot for the integrated six replicates performed at a resolution of 0.5 resulted in resulting in eight distinct cell-type clusters (Figure 2a). These clusters included synovial cells/fibroblasts (33.4% of total cells), MSC/fibroblasts (26.9%), myeloid cells (15.6%), adipocytes/endothelial cells (14.0%), vascular/visceral smooth muscle cells (5.7%), T cells (3.2%), erythrocytes (0.5%), and myelin/neuronal cells (0.5%). A heatmap of the top 10 marker genes defining each of the eight clusters is shown in Figure 2b and several representative genes with specific expression patterns defining a particular cell type are shown in Figure 2c. Importantly, there were noted differences in the proportion of cells in each cluster contributed by each sample group with a striking difference in the proportion of myeloid cells across the groups with healthy samples having only ~9% myeloid cells and significantly different compared to 18% in the disease and 25% in the treated samples (*p* < 0.05 for healthy vs. disease, healthy vs. treated, and disease vs. treated) (Table 1 and Figure 2a). When comparing Healthy with Diseased, z-statistical analysis indicated differences in cell proportions of all populations except those showing limited presence within the fat pad tissue (T cells, erythrocytes, myelin/neuronal cells). Interestingly, Diseased versus Treated comparison showed differences in cell proportions of synovial cell/fibroblasts, myeloid cells, and adipocytes/endothelial cells (Table 1).

### 3.3. Sub-Cluster Analysis of Macrophage Cells within IFP

**Figure 3:**
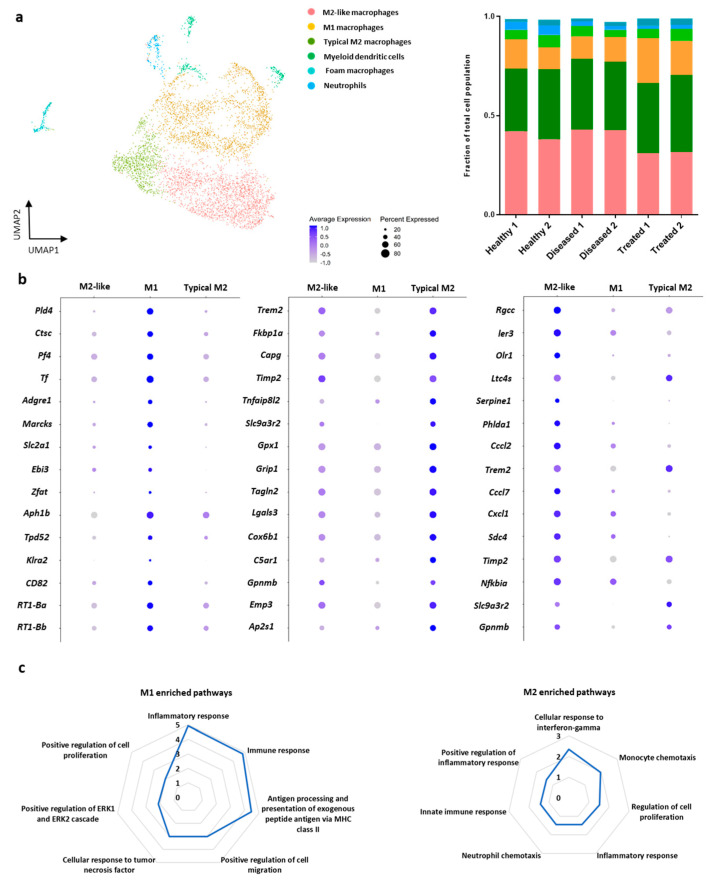
Single-cell transcriptional profile of the *CD68*^+^ myeloid cell population. (**a**) Six immune cell subsets were identified within the *CD68*^+^ cell cluster. Analysis of the marker genes for these clusters revealed three macrophages biological subtypes the M2-like macrophages, the M1 macrophages, and the typical M2 macrophages. Each point represents a single cell and separate subpopulations identified were color-coded. Cell population proportion of typical M2 macrophages relative to M1 is lowest in the diseased samples, followed by healthy and treated. Cell population proportions per sample were visualized by stacked bars plot. (**b**) Dot plot representing gene expression for macrophage polarization marker genes in all three groups tested (healthy, diseased, treated). (**c**) Enriched pathways of genes identified in M1 and M2 macrophages subclusters. Pathway analysis revealed highly enriched genes in M2 and M2-like subpopulations mostly involved in immunoregulatory pathways. In contrast, M1 enriched genes were involved in antigen processing and presentation, and cellular response to TNF/IFNγ pathways.

To explore this discrepancy in proportion of myeloid cells across the groups, we performed a sub-clustering analysis by extracting the cells from the myeloid cluster that were also positive for expression of the myeloid marker *CD68*. For these 5414 cells we re-performed normalization, scaling, and cluster identification at a resolution of 0.1 and found seven distinct clusters (Figure 3a). Analysis of the marker genes for these clusters revealed biological subtypes including M2-like macrophages (37.3% of cells), M1 macrophages (35.7%), typical M2 macrophages (15.3%), dendritic cells (5.1%), foam macrophages (2.6%), neutrophils (2.5%), and two clusters of undefined cell types (1.4%). Our M1/M2 macrophages definition was based on previous published literature including [57,58,59,60,61,62,63,64,65,66,67,68,69,70,71,72,73,74,75,76] (Table 2). We defined typical M2 by high expression of *Trem2* and showed that they have expression patterns consistent with M2-like macrophages but with even higher expression of *Slc9a3r2*, *Timp2*, and *Gpnmb* (Figure 3b and Figure S1). Furthermore, based on the overall transcriptional signatures of M1, typical M2 and M2-like macrophages we described the expression levels of individual transcripts involved separately in Diseased, Healthy, Treated groups (Figure S2). In order to determine gene expression differences between M1 and typical M2 and M2-like macrophages, we performed differential expression between those clusters. We identified 93 genes downregulated and 112 genes upregulated in the M2 and M2-like cells relative to M1 (adjusted *p* < 0.05). Overall, the significant upregulated genes for M1, typical M2, and M2-like macrophages clusters are presented in detail in Table 2. Pathway enrichment analysis indicated that downregulated genes (higher expressed in M1) were largely involved in immune responses while the genes upregulated (higher expressed in typical M2 and M2-like) were enriched in cell proliferation and chemotaxis related pathways (Figure 3c).

### 3.4. IFP-MSC Induces M2 Macrophage Polarization In Vivo

**Figure 4:**
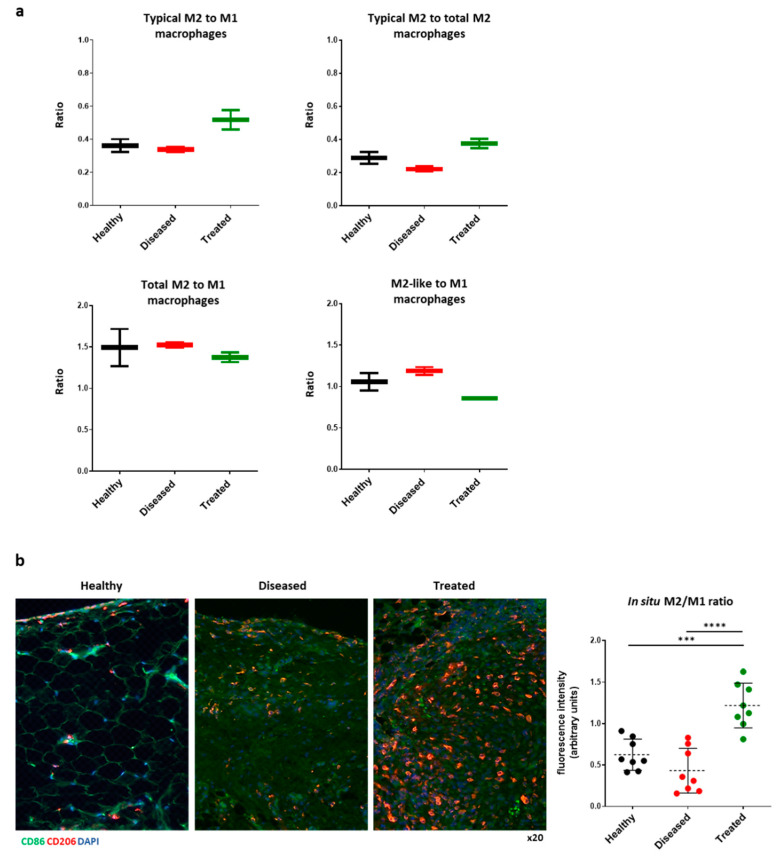
(**a**) Typical M2/M1, typical M2/total M2, total M2/M1, and M2-like/M1 ratios for each sample group based on single-cell RNA-sequencing macrophage clustering (total M2 = typical M2 + M2-like proportions). (**b**) Immunophenotyping profiling for CD86 (M1) and CD206 (M2) macrophages within the IFP tissue in all three groups tested (healthy, diseased, treated). In situ, IFP-MSC treated group showed M2/M1 significantly higher ratio than healthy and diseased groups (*** *p* < 0.001 and **** *p* < 0.0001). Statistical analysis was performed using one-way ANOVA for multiple comparison.

As in the overall clusters, the sample groups contribute different proportions of cells to each of the macrophage sub-clusters (Table 3). We observed a gradual increase in the numbers of macrophages within the fat pad when comparing healthy (1158 cells), diseased (1678 cells), and treated (1948 cells) groups. Notably, the proportion of typical M2 macrophages relative to M1 is lowest in the diseased samples (0.34), followed by healthy (0.43) and treated (0.51) (Figure 4a, upper left graph). Most importantly, IFP-MSC treatment resulted in a further M2 macrophage maturation as its evident by the increased proportion of typical M2 within the pool of M2 polarized macrophages between the diseased (0.22) and treated groups (0.38) (Figure 4a, upper right graph). Interestingly, there is no significant difference between the ratios of total M2/M1 in the investigated groups (Figure 4a, bottom left graph). Of note, M2-like/M1 ratio is higher in the Diseased group compared to Healthy and Treated groups (Figure 4a, bottom right graph). However, z-statistical analysis revealed no significant differences in cell proportions between groups except Healthy versus Treated for M2-like macrophages and Diseased versus Treated for M2-like and typical M2 macrophages (Table 3).

In situ, in addition to signs of early fibrotic changes of the IFP, we confirmed the M1 macrophage polarization 8 days after the intra-articular injection of MIA, compared with healthy knees without inflammatory induction (Figure 4b). Interestingly, in IFP-MSC group we observed mostly an M2 macrophage polarization with an M2/M1 ratio of 1.2 ± 0.3 significantly higher than healthy and diseased groups (0.6 ± 0.19, *p* < 0.01 and 0.43 ± 0.27, *p* < 0.0001, respectively). Statistical analysis was performed using one-way ANOVA for multiple comparisons.

## 4. Discussion

Diseases of the joint involve a complex interplay between host tissues and resident immune cells. The IFP and synovium are considered a single functional unit [1], exhibiting a tight molecular crosstalk implicated in maintaining joint homeostasis as well as the onset of inflammatory joint disease [2,3,4]. We and others have shown that biologically the IFP participates in the pathogenesis and progression of various pathologies within the joint, with a heterogeneous cellular composition consisting of adipocytes, fibroblasts, and in smaller quantities resident mast cells, lymphocytes, and perhaps most importantly macrophages [25,26,77]. In the present study, we dissected by single-cell RNA-sequencing the IFP cellular heterogeneity and associated transcriptional programs to help elucidate the dynamic cellular programs involved in homeostasis and potentially synovitis/IFP fibrosis. We described the IFP cellular alterations that are associated with MSC treatment of an inflamed knee characterized by synovitis and fibrosis, with focus on macrophage phenotypic polarization. This approach may direct new cellular therapies for early OA treatment, including the development of post-traumatic OA following injuries such as anterior cruciate ligament (ACL) disruption. Multiple studies support the notion that the IFP and synovium undergo recurrent cycles of inflammation resulting in progressive IFP fibrotic changes that are involved in the onset and progression of inflammatory joint disease [2,3,4,5,6,7,8,9,10].

First of all, similar to our previous studies [26,40,78] we found neither clinical nor histological signs of xeno-rejection of the human cells by the host which supports the notion of the immunoevasive properties of MSC [79]. MIA intra-articular injection represent a chemical induced inflammatory joint disease model that results in synovitis inflammation/IFP fibrosis, chondrocyte cell death, and functional impairment [80]. On this basis, we acknowledge the artificial nature of MIA model, however it is a valuable model that creates robust and reproducible synovitis/IFP fibrosis and early-stage OA pain phenotypes [43,81]. Therefore, due to the short-term (8 days in total) duration of our in vivo investigation, the MIA model was ideal to study IFP cellular heterogeneity and associated transcriptional programs at the onset of inflammatory joint disease. 

Similar to our previous reports [26,40], we confirmed that intra-articular injection of MIA into the knee joint rapidly triggers an inflammatory response leading to synovitis and IFP fibrosis, whereas a subsequent single IFP-MSC intra-articular injection largely reverses those tissues responses. Eight transcriptomically distinct cell populations were identified across all three groups (healthy, diseased, treated) after single-cell sequencing of the dissociated IFP tissue. We clearly demonstrated that these structural transitional changes between diseased and treated animals were reflected in the cell population proportions, with IFP-MSC infused fat pads increasing the proportion of macrophages making up the IFP. A similar single-cell RNA-sequencing study by Stephenson et al. in rheumatoid arthritis (RA) patients revealed 13 transcriptomically distinct cell subsets within the synovium. Various immune-related cell populations such as macrophages, dendritic cells, CD4+/CD8+ T cells, B cells, and NK cells were detected [82]. Similar to our findings, RA patients showed an increased proportion of macrophages compared to other cell types. Furthermore, it is well known that macrophage-like resident synoviocytes exist within the intimal synovial lining and have pro-inflammatory tendencies that play a key role in both autoimmune and OA disease development [83,84]. In the present study, the IFP was carefully dissected from surrounding tissues, including the synovium, in order to evaluate its distinct involvement in progression of joint inflammation. Taken together, we propose that not only synovium- but also IFP-derived resident macrophages are present in homeostasis (healthy), and together with infiltrating macrophages are strongly involved in both the progression and resolution of knee joint inflammation through phenotypic polarization towards pro- and anti-inflammatory states.

Previous studies have demonstrated that macrophages are not only permanently resident but are also activated by a variety of interleukins and interferons secreted from other resident and infiltrating immune cells and adipocytes within the IFP [3,85]. Upon M1 macrophage activation, the IFP begins secreting vast amounts of pro-inflammatory cytokines, pro-fibrotic mediators such as CTGF, catabolic factors, and adipokines that contribute to joint pathologies [3]. In the present study, further transcriptional dissection of the *CD68*^+^ myeloid population revealed six immune cell subsets including macrophages, dendritic cells, and neutrophils that possess distinct transcriptional signatures and are likely to contribute to inflammatory disease etiology. Most importantly, we report both M1 and M2 macrophage polarization within IFP tissue in all three groups tested (healthy, diseased, treated). Interestingly, the M2 macrophage subset consisted of two M2 alternative activation variants, one showing transitional transcriptional profile between M2 and M1, and one showing typical M2 macrophage polarization. Specifically, we defined typical M2 by high expression of *Trem2* among other M2 polarization related genes, as multiple studies showed that *Trem2* is a typical M2 macrophage marker [86,87]. These findings are supported by the notion that macrophages may encompass a broad spectrum of phenotypes in vivo beyond the conventionally recognized pro-inflammatory M1 and anti-inflammatory M2 states [28,57]. Relatedly, Mould et al. based on a similar high-resolution dissection identified transcriptionally distinct macrophage subsets involved in homeostasis, acute inflammation, and resolving inflammation in the lung alveolar tissue [88]. In the present study, pathway and gene-set enrichment analysis revealed hierarchical relationships between M2 and M1 macrophage subpopulations that were consistent with transcriptomic similarities. However, pathway analysis suggested heterogeneous pathway activation with highly enriched genes in M2 and M2-like subpopulations mostly involved in immunoregulatory pathways. Importantly, studies showed that M2 macrophages possess immunoregulatory functions via the secretion of anti-inflammatory molecules including IL-10 and IL-1RA, and chondroprotective functions via the secretion of pro-chondrogenic factors, such as TGF-β [89,90]. On this basis, M2 macrophages polarization presents a significant potential for OA treatments. Similar to previous reports, M1 enriched genes were involved in antigen processing and presentation, and cellular response to TNF/IFNγ pathways [57]. These pathway analyses indicate that the M1 to M2 polarization ratios identified in all three groups (healthy, diseased, treated) are directly related to joint inflammation status and subsequently joint disease progression.

Upon IFP-MSC infusion (treated group) we observed not only an increased overall proportion of macrophages within the IFP but most importantly a shift of M2-like macrophages towards a typical M2 phenotype compared to the diseased and healthy groups. This transcriptional program alteration suggests an IFP microenvironment shift from a pro-inflammatory state in the diseased synovitis group to an immunomodulatory status in the IFP-MSC treated group. On this basis, immunophenotypically the IFP-MSC treated IFP tissue showed a significantly higher presence of M2 polarized macrophages to M1 compared to the diseased and healthy groups. Taken together, the data suggest a plausible mechanistic explanation of our previous findings [26,40] indicating that the transient engraftment of IFP-MSC on the synovium results in the reversal of active synovitis and IFP fibrosis in vivo. According to previous studies, dysregulation of the M1/M2 phenotypic balance of IFP macrophages may account for triggering and further progression of OA by helping to maintain a sustained low-grade inflammatory state [28,29]. Additional pre-clinical studies to confirm this important finding could direct clinical trials of patients with knee OA.

The information gathered with this study helps understand better the cellular crosstalk implicated in maintaining joint homeostasis as well as the onset of inflammatory joint disease. However, this study has some limitations that deserve consideration. Our previous studies using an MIA rat model indicated that a single intra-articular injection of 500,000 IFP-MSC results in synovitis/IFP fibrosis reversal on day 4 post-infusion [26,40,78]. The same experimental strategy was deployed in the present study to investigate cellular heterogeneity. However, further assessment of the MSC therapeutic dosage infused and IFP tissue evaluation at different timepoints post-infusion (>4 days) could result in better understanding of M2/M1 macrophages polarization alterations. On this basis, prolonged investigation of IFP tissue M2/M1 macrophages polarization alterations could define the ‘terminal’ polarization of M2-like subpopulation towards M2 macrophages. On another note, we acknowledge that the use of another acute monoarthritis model could better recapitulate the events of OA initiation and progression. Technical considerations from the single cell RNAseq are also limitations including sample size in each group, exploration of the phenotypes across sexes, and investigation of various timepoints.

## 5. Conclusions

In summary, application of single-cell RNA-sequencing technology to the rat IFP yielded a comprehensive picture of the tissue cellular heterogeneity and led to the identification of M2 and M1 polarized macrophage subsets with variable ratios between healthy, diseased, and IFP-MSC treated groups. Our results confirm that macrophages exist in the IFP during acute synovitis/IFP inflammation progression that can be shifted towards a more immunomodulatory polarization status after a single IFP-MSC intra-articular therapy. These findings suggest possible novel therapeutic approaches for joint pathologies that modulate macrophage populations away from inflammatory phenotypes in the IFP.

## Figures and Tables

**Table 1:** Overall cell count and proportion in each defined cluster for each sample group.

	Healthy		Diseased		Treated		Differences between Groups (z-Statistics)		
Cluster	Cell Count	Proportion	Cell Count	Proportion	Cell Count	Proportion	Healthy versus Diseased	Healthy versus Treated	Diseased versus Treated
Synovial cell/Fibroblasts	6871	0.375	3461	0.315	2513	0.276	* (2.280 × 10^−9^)	* (6.835 × 10^−19^)	* (1.248 × 10^−3^)
MSC/Fibroblasts	6426	0.351	2166	0.197	1750	0.192	* (1.304 × 10^−40^)	* (1.125 × 10^−36^)	ns
Myeloid cells	1704	0.093	2017	0.183	2287	0.251	* (6.350 × 10^−15^)	* (5.088 × 10^−37^)	* (9.325 × 10^−8^)
Adipocytes/Endothelial cells	2202	0.120	1981	0.180	1214	0.133	* (7.036 × 10^−8^)	ns	* (6.052 × 10^−4^)
Vascular/Visceral Smooth Muscle cells	552	0.030	849	0.077	793	0.087	* (4.302 × 10^−4^)	* (5.531 × 10^−5^)	ns
T cells	452	0.025	362	0.033	449	0.049	ns	ns	ns
Erythrocytes	51	0.003	99	0.009	45	0.005	ns	ns	ns
Myelin/Neuronal cells	72	0.004	65	0.006	51	0.006	ns	ns	ns

* *p*< 0.05 (two proportion z-test); ns: non-significant.

**Table 2:** Macrophage polarization: different gene signatures in M1, typical M2, and M2-like macrophages.

Clusters					
M1 Macrophages	References	Typical M2 Macrophages	References	M2-like Macrophages	References
*Pld4*	[59]	*Trem2*	[57]	*Rgcc*	[58]
*Ctsc*	[60]	*Fkbp1a*	[57]	*Ier3*	[72]
*Pf4*	[61]	*Capg*	[57]	*Olr1*	[73]
*Tf*	[62]	*Timp2*	[68]	*Ltc4s*	[57]
*Adgre1*	[63]	*Tnfaip8l2*	[69]	*Serpine1*	[74]
*Marcks*	[64]	*Slc9a3r2*	[57]	*Phlda1*	[76]
*Slc2a1*	[65]	*Gpx1*	[57]	*Ccl2*	[75]
*Ebi3*	[57]	*Crip1*	[57]	*Trem2*	[57]
*Zfat*	[57]	*Tagln2*	[57]	*Ccl7*	[57]
*Aph1b*	[57]	*Lgals3*	[57]	*Cxcl1*	[57]
*Tpd52*	[57]	*Cox6b1*	[57]	*Sdc4*	[57]
*Klra2*	[57]	*C5ar1*	[70]	*Timp2*	[68]
*CD82*	[57]	*Gpnmb*	[71]	*Nfkbia*	[57]
*RT1-Ba*	[66]	*Emp3*	[57]	*Slc9a3r2*	[57]
*RT1-Bb*	[67]	*Ap2s1*	[57]	*Gpnmb*	[71]

**Table 3:** Cell count and proportion in each defined myeloid cell population for each sample group.

	Healthy		Diseased		Treated		Differences between Groups (z-Statistics)		
Cluster	Cell Count	Proportion	Cell Count	Proportion	Cell Count	Proportion	Healthy versus Diseased	Healthy versus Treated	Diseased versus Treated
M2-like macrophages	535	0.399	797	0.427	690	0.313	ns	* (1.92 × 10^−3^)	* (6.55 × 10^−6^)
M1 macrophages	452	0.337	659	0.353	821	0.372	ns	ns	ns
Typical M2 macrophages	171	0.127	222	0.119	437	0.198	ns	ns	* (1.75 × 10^−2^)
Dendritic cells	74	0.055	82	0.044	119	0.054	ns	ns	ns
Foam macrophages	60	0.045	41	0.022	39	0.018	ns	ns	ns
Neutrophils	30	0.022	32	0.017	75	0.034	ns	ns	ns

* *p* < 0.05 (two proportion z-test); ns: non-significant.

## Data Availability

The data presented in this study are available in the article or supplementary material.

## Supplementary Materials

The following are available online at https://www.mdpi.com/article/10.3390/bioengineering8110166/s1, Figure S1: Gene localization plots for macrophage polarization marker genes related to distinct M1, M2-like and typical M2 macrophages subsets, overlaid on the UMAP visualization dot plot. Gene expression levels depicted from gray (low) to purple (high). Figure S2: Expression levels of individual transcripts consisting of M1, typical M2, and M2-like macrophage signatures in Diseased (D), Treated (T), and Healthy (H) groups.
