# Supplementary material for: Single-Cell RNA-Sequencing Identifies Infrapatellar Fat Pad Macrophage Polarization in Acute Synovitis/Fat Pad Fibrosis and Cell Therapy
Source: Bioengineering (Basel). 2021 Oct 29;8(11):166. doi: 10.3390/bioengineering8110166 (PMC8615266; doi:10.3390/bioengineering8110166)
Supplement: Supplementary file 1 [file bioengineering-08-00166-s001.zip › bioengineering-1395622-supplementary.pdf]

bioengineering

Supplementary Materials and methods: Single-Cell RNA-Sequencing Identifies Infrapatellar Fat Pad Macrophage Polarization in Acute Synovitis/Fat Pad Fibrosis and Cell Therapy

Dimitrios Kouroupis, Thomas M. Best, Lee D. Kaplan, Diego Correa, Anthony J. Griswold

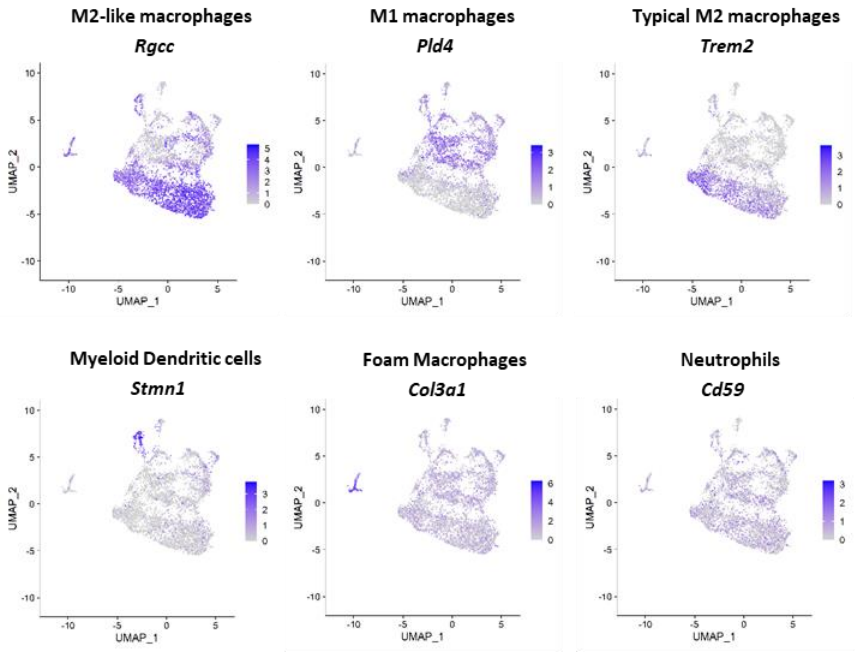

Figure S1. Gene localization plots for macrophage polarization marker genes related to distinct M1, M2-like and typical M2 macrophages subsets, overlaid on the UMAP visualization dot plot. Gene expression levels depicted from gray (low) to purple (high).

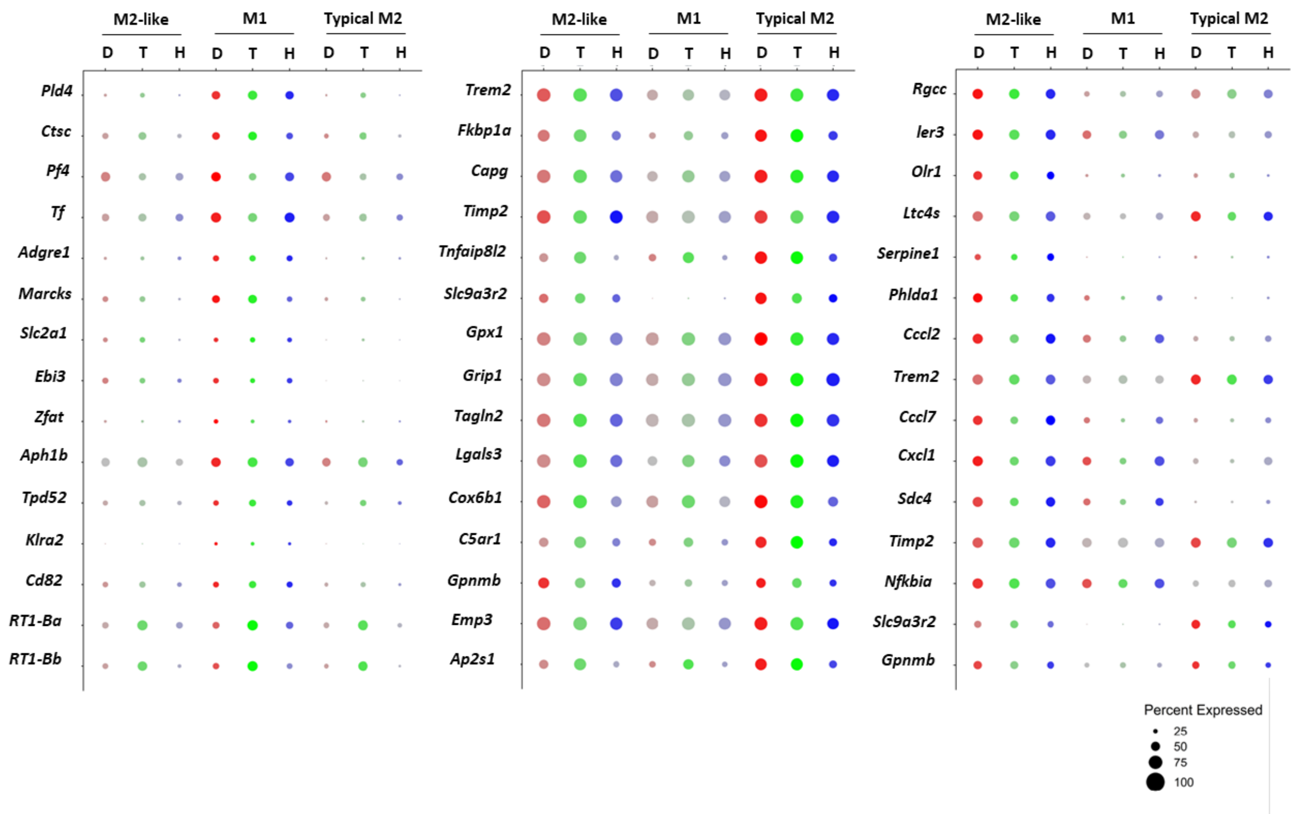

Figure S2. Expression levels of individual transcripts consisting M1, typical M2, and M2-like macrophage signatures in Diseased (D), Treated (T), and Healthy (H) groups.

© 2021 by the author. Licensee MDPI, Basel, Switzerland. This article is an open access article distributed under the terms and conditions of the Creative Commons Attribution (CC BY) license (https://creativecommons.org/licenses/by/4.0/).
